# A Comprehensive Analysis of Post-partum Depression Risk Factors: The Role of Socio-Demographic, Individual, Relational, and Delivery Characteristics

**DOI:** 10.3389/fpubh.2019.00295

**Published:** 2019-10-24

**Authors:** Martina Smorti, Lucia Ponti, Federica Pancetti

**Affiliations:** ^1^Department of Surgical, Medical and Molecular Pathology and Critical Care Medicine, University of Pisa, Pisa, Italy; ^2^Department of Educations, Languages, Intercultures, Literatures and Psychology, University of Florence, Florence, Italy; ^3^Division of Obstetrics and Gynaecology, Department of Clinical and Experimental Medicine, University of Pisa, Pisa, Italy

**Keywords:** postnatal depression, childbirth, maternal mental health, risk factor, nulliparous women

## Abstract

Postpartum depression is a common and complex phenomenon that can cause relevant negative outcomes for children, women and families. Existing literature highlights a wide range of risk factors. The main focus of this paper is to jointly investigate different types of risk factors (socio-demographic, psychopathological, relational, and related to labor and birth experience) in post-partum depression onset in women during first-child pregnancy, identifying which of these are the most important predictors. A cohort longitudinal study was conducted on 161 Italian nulliparous low-risk women (*M*_*age*_ = 31.63; *SD* = 4.88) without elective cesarean. Data was collected at three different times: Socio-demographic, prenatal anxiety and depression, and quality of close relationship network (with mother, father and partner, and the prenatal attachment to child) were assessed at T1 (week 31–32 of gestation); clinical data on labor and childbirth (mode and typology of delivery, duration of labor, duration of eventual administration of epidural analgesia, and child's APGAR index at birth) were registered at T2 (the day of childbirth); and the degree of post-natal depression symptomatology was measured at T3 (1 month after birth). Postpartum depression is associated with several risk factors (woman's age, woman's prenatal psychopathological characteristics, the level of prenatal attachment to child, the quality of romantic relationship, and some clinical delivery difficulties). Overall, the level of prenatal attachment to child was the most important predictor of post-partum depression. These findings emphasize the very important role of prenatal attachment for the onset of postpartum depression and the need to promote adequate and targeted prevention interventions. Limitations, strengths, and theoretical and clinical implications are discussed.

## Introduction

For a woman, the gestation of her first child has been identified as a central life event (1). From a psychological perspective, in fact, the pregnancy of the first baby involves the transition to motherhood, a major developmental period with important implications for mothers, for the infant-mother relationship, and the infant's development (2). During the first pregnancy, a woman's maternal identity develops through the reorganization of mental self-representation and the elaboration of other significant relationships (3, 4). The woman's mental self-representation enriches with the maternal component, thus leading her to review the relationship with her own mother; the mental couple image gradually modifies with integration of the family image, and the marital relationship is reorganized with the parental component. With the birth of the first child, the quality of the couple relationship may undergo temporary changes that are influenced by the ability of the parents to adapt to new needs (5).

The transition to parenthood (transition to parenthood—TTP) has often been associated with marital crisis, and the premise in literature was that parenthood creates serious individual and relationship distress (6, 7). Parenting can be an improvement factor for some couple relationships; however, it can also be disruptive and increase problems (8). While some couples may develop new skills in resolving difficulties, others find themselves running aground trying to develop these skills (6).

All the above physical, psychological, and relational changes that occur during the perinatal period may increase the risk for maternal emotional vulnerability, such as depressed emotions. The DSM-5 proposes the term “peripartum onset” as a major depressive episode during pregnancy or in the weeks or months following delivery (9). This condition is characterized by sad mood, anxiety, irritability, lack of positive emotions, loss of pleasure, interests and energy, decreased appetite, inability to cope, fear of hurting self and baby, and suicidal thoughts (10, 11). Both anxiety and depression can occur in the perinatal period (up to 1 year after delivery) (9) and these conditions present high rates of co-morbidity (12). The first weeks immediately after childbirth are the most critical (13), and although the increased vulnerability continues for the following 6 months (14–16), post-partum depression (PPD) generally occurs within the first month after delivery (9).

This vulnerability is higher for primiparous mothers, who present an increased risk for depression in the postpartum period (12, 17, 18), especially in the first 90 days after delivery (19). Although contrasting results emerged about the prevalence of postpartum depression in relation to parity (20, 21), studies conducted in the European context showed that the prevalence for PPD in primiparas was 11.8 vs. 8.6% in multiparas (21).

In fact, maternal inexperience leads new mothers to have greater difficulty in early interactions with their children, and research has reported that the effect of maternal depression is greater in nulliparas compared to multiparas (22).

Although from a psychiatric perspective postpartum depression is no longer classified as a distinct entity, from a psychological perspective, depression occurring during the postpartum period has particular relevance in a woman's life.

In fact, it enhances the risk of a multitude of negative consequences for children, women, and families, including poor infant physical health and more frequent sickness, and physiological, psychological, emotional, and psychomotor delays during infancy and early childhood (23).

Moreover, PPD can negatively affect the ability and the availability of women to adequately take care of their children. Henderson et al. (24) have shown the important negative outcomes that PPD can have on mother-infant interactions, the child's growth, and the tendency to quit breastfeeding earlier. Moreover, children of mothers with PPD tend to establish insecure attachment bonds and develop social difficulties with peers (25, 26).

Because of these relevant consequences, it is important to identify the risk factors that can be involved in the development of PPD in first-time mothers.

Several factors, both internal and external, have been found to be related to PPD, and it is plausible that a complex interplay of these can be the cause of greater vulnerability (27), especially in women during their first pregnancy, compared to those who already have a child. Alongside psychological risk factors, such as a lifetime history of depression, and a presence of antenatal depression and prenatal anxiety (28, 29), many authors have shown that relational variables constitute significant risk factors for post-partum depression development. Problems in maternal and romantic relationships, such as marital instability, and low level of maternal (30, 31) and marital support (32) have been found to be linked with PPD. Priel and Besser (33) have also found that antenatal attachment was closely linked to PPD. A higher level of antenatal attachment to child predicted a lower level of depressive symptomatology after birth. The presence of stressful life events, or the lack of social support from peer and health professionals, can foster the subsequent development of PPD (34–36).

There are other clinical aspects linked to pregnancy and delivery that are associated to the PPD condition. A complicated labor and birth characterized by longer length of labor and greater pain, or medical intervention during delivery, can result in negative consequences, varying from maternal distress to PPD (31, 37). Given that the nulliparous tend to be less self-confident in the maternal role, and that being less self-confident has been associated with postpartum depression (38), labor and delivery complications can be particularly difficult for first-time mothers.

Finally, some socio-demographic characteristics, such as a young age, or low level of education, or low income, may be considered linked to a higher probability of developing PPD (39, 40).

Despite the relevance of these risk factors, most studies have focused on psychopathology and social network aspects linked to maternal and romance relationships, and less attention has been directed to the exploration of these factors jointly.

The aims of this study were: (1) to confirm previous results exploring the role that several sets of variables, such as socio-demographic, individual, relational, and related to delivery characteristics, separately considered, play as risk factors for the onset of postpartum depression; and (2) to verify which, among the above risk factors, have a more significant influence when they are considered together.

In accordance with literature, it was hypothesized that: (1) young age, low level of education, low employment status, and not planned pregnancy, could positively predict levels of PPD; (2) prenatal anxiety and depression positively predict PPD; (3) an affectionate prenatal attachment, and a good quality romantic and parental relationship negatively predict PPD; (4) a more complicated labor (in terms of duration and duration of epidural and oxytocin administration), the modality of delivery (cesarean section vs. vaginal birth) and a worse index of newborn well-being (in terms of lower Apgar score) positively predict PPD. No hypotheses were developed about the strongest predictor for PPD.

## Materials and Methods

### Procedure and Participants

The study was conducted in accordance with the guidelines for the ethical treatment of human participants of the Italian Psychological Association. The Ethical Committee of Azienda USL 4 Prato, Italy, had previously approved the study (no 780/2013). Data were collected during 2014 in the maternity ward of a public hospital of the metropolitan area of Prato (Italy), a unit with about 1,130 deliveries per year (69% Italian women), from January to December 2014, during delivery preparation courses organized for pregnant women (>30 weeks of gestation).

A cohort longitudinal study was carried out. Data were collected at three different time points: (1) 31–32 week of gestation; (2) the day of delivery; and (3) 1 month after childbirth.

Inclusion criteria were: Italian women, age >18 years, physically and psychologically healthy nulliparous women with singleton low-risk pregnancies, gestational age >31 weeks. Exclusion criteria were: twin pregnancy, maternal pathologies during pregnancy, fetal pathologies, the presence of depressive pathologies documented in clinical records, and planned elective cesarean. Planned elective cesarean was an exclusion criterium because we were interested in examining the roles of labor and delivery as predictors of PPD. Therefore, we excluded from the study women who underwent planned elective cesarean, but not those who experienced emergency cesarean after labor.

The participants (*n* = 191) were informed about the aims of the study and signed a written informed consent form. They could withdraw from participation at any time. Ninety-four percent of the women who were contacted consented to participate in the survey (*n* = 179) and, of them, 90% completed the entire follow-up (Time 1, 2, and 3). At T1 we recruited 179 women, but by T3 we lost 18 women who did not return the completed questionnaire. The final sample consisted of 161 nulliparous pregnant women aged 18–42 years (*M* = 31.63, *SD* = 4.88). Our sample is representative regarding both size and age of the general population of women giving birth in Prato that meet our inclusion criteria.

### Measure

At time 1, all participants received a battery of questionnaires for the collection of socio-demographic, clinical, psychological and relational data. In particular:

#### Socio-Demographic and Clinical Measures

Participants provided their age, educational level, work status, marital status, information about the number of years of their couple relationship, and information about planned or not pregnancy.

#### Psychological Measures

Participants were asked to complete psychological questionnaires to assess psychopathological characteristics. To assess the women's anxiety level, the State Anxiety Inventory (STAI_Y2) (41, 42) was used. This questionnaire is the most widely used measure of anxiety during pregnancy, especially in association with postnatal depression (43). The STAI_Y2 is a 20-item self-report questionnaire asking to report how often the anxiety state was experienced. Responses were rated on a 4-point Likert scale, from 1 (never) to 4 (very often) The total score is obtained by summing all items, after some items are overturned, and can range from 20 to 80. A high score indicates a high level of anxiety. For the current study, Cronbach's alpha was 0.90.

To detect the level of women's depression, the Beck Depression Inventory (BDI) (44, 45) was used. The BDI is a 21-item self-report inventory used for measuring the severity of symptoms. Each item had a set of four responses ranging in intensity from 0 to 3. The total score is obtained by summing all items and can range from 0 to 63. High scores indicate high depressive symptomatology. In the present sample, Cronbach's value was 0.84.

#### Relational Measures

All women were asked to complete four questionnaires assessing the quality of their close relationship network, with their mother, father and partner, and the level of their prenatal attachment to child. In particular, the quality of women's relationships with mothers and fathers was assessed using the Parental Bonding Instrument (PBI) (46, 47). The PBI consists of two parallel versions of 21 items, ranging from 0 (Very likely) to 3 (Very unlikely), which assessed three dimensions: Care, Encouragement toward autonomy, and Overprotection. In the present sample, Cronbach's values for the paternal version were 0.98, 0.98, and 0.96 for Care, Encouragement toward autonomy, and Overprotection, respectively. For the maternal version, Cronbach's values for Care, Encouragement toward autonomy, and Overprotection were 0.98, 0.96, and 0.87, respectively. In this study, we used a global dimension of the relationship quality, summing the above three dimensions, according to the procedure described in the results section. The total score for PBI is obtained by summing all items and can range from 0 to 63. High scores on this dimension indicate that the women perceive a good quality of their maternal and paternal relationships.

The quality of the women's romantic relationships was assessed using the Romance Qualities Scale (RQS) (48). The RQS is a 22-item self-report instrument, ranging from 1 (Absolutely false) to 5 (Absolutely true), which assesses five main qualitative dimensions of the relationship with partner (companionship, conflict, help, security and closeness) and a global score of the romantic relationship quality. The total score is obtained by summing all items and can range from 22 to 110. High scores on this dimension indicate that women perceive a good quality of their romantic relationships. In the present sample, Cronbach's value was 0.84.

The Prenatal Attachment Inventory (PAI) (49, 50) was used to measure the mother's attachment bond to her child during pregnancy. The PAI is a self-report questionnaire with 21 items from 1 (Almost never) to 4 (Almost always). The total score is obtained by summing all items and can range from 21 to 84. High scores indicate a good quality of prenatal attachment bond. For the present sample, the Cronbach's alpha was 0.93.

At time 2, clinical information regarding labor, delivery and birth outcomes was extracted from hospital records after childbirth. In particular:

Labor measures including three indices: (a) modality of labor (induced vs. spontaneous); (b) duration of labor in hours; (c) administration of epidural analgesia in hours (no analgesia administration = 0).

Mode of delivery, recorded according to category: vaginal (natural and operative vaginal delivery) vs. emergency cesarean delivery.

Birth outcomes, assessed via APGAR scores at 1 min. APGAR score index at birth was determined by evaluating the newborn baby on: color, heart rate, reflexes, muscle tone, respiration). Scores ≤3 are generally regarded as critically low, 4–6 fairly low, and 7–10 generally normal.

At time 3, women were requested to fill out a psychological questionnaire to assess the degree of postnatal depression symptomatology. Diagnosing depression in post-partum may be particularly challenging due to the overlap of diagnostic depressive symptoms with those of a normal post-partum period for women (e.g., fatigue, decreased libido, and sleep or appetite change). Nevertheless, as documented by several authors, the Edinburgh Postnatal Depression Scale (EPDS) (51, 52), originally devised for the identification of postpartum depression disorders, allows us to measure affective aspects rather than physical symptoms of depression that may be affected by the perinatal period (52). The EPDS is a self-report questionnaire consisting of 10 items ranging from 0 to 3, according to increasing severity of the symptom. The total score is obtained by summing all items and can range from 0 to 30 with higher scores on this scale indicating higher levels of postnatal depression symptomatology. For the current sample, Cronbach's alpha was 0.88.

### Data Analysis

Data were analyzed using SPSS version 24 (2017). Frequency, means, standard deviation, and bivariate correlation were calculated for all variables. To determine the relationship between predictor variables and postpartum depression, linear regression analyses were separately undertaken for each set of risk factor variables considered.

Regarding parental relationships, we were interested in creating an aggregate score of the quality of maternal and paternal relationship. To verify the possibility to use a single score for the quality of maternal and paternal relationships to include in the regression analysis, two factorial analyses with the three dimensions of the PBI were conducted, separately for the mother and father versions.

Subsequently, to explore the stronger risk factors, a linear regression (stepwise method) was conducted with post-partum depression as the dependent variable, and the significant risk factors were entered as predictors. An alpha level of 0.05 was used for all statistical tests.

## Results

All the women had a middle or high socioeconomic level; 87% had a high school diploma or bachelor's degree (13% of women had a secondary school diploma, 54% had a high school diploma, and 33% a bachelor's degree or more) and 81.4% of the women had a job. Regarding marital status, 100% of participants lived with their partners, and 59.1% were married. The length of romantic relationships ranged from 1 to 17 years (*M* = 6.25, *SD* = 3.81). Pregnancy was planned in 82.6%.

The regression analysis with socio-demographic characteristics as independent variables and the score of PPD as a dependent variable showed that the model composed by age, length of the romantic relationship, level of education (dummy variable: 1 = high school or university degree; 0 = middle school or elementary school degree), employment status (dummy variable: 1 = employed; 0 = unemployed), and planned pregnancy (dummy variable = 1 = non-planned; 0 = planned) explained only 6% of the variance (Table 1). Specifically, data showed that the severity of PPD was positively affected by the age of women. On the contrary, the length of the relationship with partner, level of education, employment status, and planned pregnancy did not significantly affect the level of PPD.

**Table 1:** Summary of the linear regression analysis with socio-demographical characteristics as independent variables for PPD score.

**Variables**	***B***	***SE***	***B St*.**	***t***	***p***	***95% CI***	
Age	0.309	0.095	0.299	3.237	0.001	0.120	0.497
Length of relationship	−0.146	0.117	−0.111	−1.250	0.213	−0.377	0.085
Educational level	−1.129	1.16	−0.076	−0.969	0.334	−3.431	1.173
Employment status	0.538	1.03	0.042	0.524	0.601	−1.490	2.566
Planned pregnancy	−0.325	1.14	−0.025	−0.285	0.776	−2.576	1.926

### Psychopathological Characteristics and PPD Condition

Table 2 shows the means, standard deviations, and pair-wise correlation coefficients for the two dimensions of psychopathological antenatal characteristics (anxiety and depression) and the PPD condition. A high level of PPD was associated with a high level of prenatal anxiety and depression. Moreover, prenatal anxiety and depression were significantly and positively correlated.

**Table 2:** Descriptive statistics, correlations and summary of the linear regression analysis with psychopathological characteristics as independent variables for PPD score.

		***M***		***SD***	**1**	**2**	**3**
1. Anxiety		35.22		8.01	–	0.63^**^	0.58^**^
2. Depression		9.30		5.15		–	0.54^**^
3. Postpartum depression		7.53		5.03			–
**Variables**	***B***	***SE***	***B St***.	***t***	***p***	***95% CI***	
Anxiety	0.253	0.050	0.403	5.042	0.000	0.154	0.353
Depression	0.278	0.078	0.285	3.562	0.000	0.124	0.432

** *p < 0.01*.

The linear regression performed with prenatal anxiety and depression on PPD score explained 38% of the variance (see Table 2). Both these variables positively affect the level of PPD.

### Close Relationships Network and PPD Condition

To obtain a global score of the women's relationship quality with their mothers and fathers, two factor analyses were conducted with the three dimensions of the PBI, for mother and father, separately. Because correlation analyses between the three dimensions of the PBI in relation to the maternal and paternal versions showed that the Overprotection dimension is negatively correlated with Care (maternal: *r* = −0.77; paternal: *r* = −0.58) and Encouragement toward autonomy (maternal: *r* = −0.89; paternal: *r* = −0.93) dimensions, the Overprotection score was reversed before carrying out the factorial analyses to obtain saturations of the same mark on the hypothetical common factor (the single score of the PBI measure).

The results of the factor analyses showed that the dimensions of the PBI (Care, Encouragement toward autonomy and Low Overprotection) loaded into a single factor for both the maternal and paternal versions. Specifically, regarding the maternal version, the three dimensions accounted for 86.15% of total variance. Regarding the paternal version, the three dimensions accounted for 83.99% of total variance.

In conclusion, both for the mother and father versions, high scores on this dimension express warm, positive and supportive parental behaviors, reflecting a good quality of parental relationships.

In Table 3, the descriptive statistics of the close relationship variables and their pair-wise correlation coefficients with PPD are shown. The level of PPD was negatively and significantly correlated with the women's relationship quality with their mothers, fathers and romantic partners, and their prenatal attachment to child. Moreover, the prenatal attachment was positively correlated with the quality of the three close relationships (mother, father and partner). Finally, the quality of maternal relationship was significantly and positively correlated with the quality of paternal relationship. Given the high correlation between maternal and paternal relationships (*r* = 0.87), in order to avoid multicollinearity problems, a single score of these aspects was calculated. In other words, we composed a score of parental relationship by calculating the mean of the two scores.

**Table 3:** Descriptive statistics, correlations and summary of the linear regression analysis with the quality of parental, romantic and prenatal relationships as independent variables for PPD score.

	***M***	***SD***	**1**	**2**	**3**	**4**	**5**
1. Mother relationship	31.44	9.65	–	0.87^**^	0.14	0.25^**^	−0.37 ^**^
2. Father relationship	31.45	10.00		–	0.17^*^	0.23^**^	−0.37^**^
3. Partner relationship	77.25	6.32			–	0.26^**^	−0.39^**^
4. Prenatal attachment	61.94	10.17				–	−0.66^**^
5. Postpartum depression	7.53	5.03					–
**Variables**	***B***	***SE***	***B St***.	***t***	***p***	***95% CI***	
Parental relationship	−0.115	0.030	−0.216	−3.80	0.000	−0.174	−0.055
Partner relationship	−0.165	0.046	−0.207	−3.624	0.000	−0.255	−0.075
Prenatal attachment	−0.273	0.039	−0.551	−9.467	0.000	−0.330	−0.216

** p < 0.01,

* *p < 0.05*.

The linear regression showed that the model composed by parental relationship, romantic relationship and prenatal attachment explained 52% of the variance (see Table 3). The quality of parental and romantic relationship and prenatal attachment to child seems to positively affect the level of PPD.

### Labor, Delivery, and Birth Outcome Characteristics and PPD Condition

87.6% of the women had spontaneous labor, and in the remaining 12.4% labor was induced. 89.4% of women had vaginal deliveries, and 10.6% had emergency cesarean deliveries. Significant differences emerged with respect to PPD regarding the mode of labor [spontaneous vs. induced: *t*_(159)_ = −5.311; *p* = 0.000] and mode [vaginal vs. cesarean: *t*_(159)_ = 7.429; *p* = 0.000] of delivery. Specifically, women who had an induced labor showed a higher level of PPD than women who had a spontaneous one. In the same way, women who had a cesarean delivery reported a higher level of PPD than women with vaginal delivery.

Table 4 presents the descriptive statistics and correlation coefficients of all continuous delivery variables.

**Table 4:** Descriptive statistics, correlations and summary of the linear regression analysis with the delivery characteristics as independent variables for PPD score.

		***M***	***SD***	**1**	**2**	**3**	**4**
1. Duration of labor		7.40	2.22	–	0.66^**^	0.04	0.51^**^
2. Epidural analgesia		1.87	2.88		–	−0.01	0.64^**^
3. APGAR index		8.79	0.862			–	0.11
4. Postpartum depression		7.53	5.03				–
**Variables**	***B***	***SE***	***B St***.	***t***	***p***	***95% CI***	
Modality of labor	−0.356	1.147	−0.023	−0.310	0.757	−2.623	1.910
Typology of delivery	−3.167	1.226	−0.194	−2.582	0.011	−5.589	−0.744
Duration of labor	0.265	0.180	0.117	1.471	0.143	−0.091	0.621
Epidural analgesia	0.814	0.165	0.465	4.938	0.000	0.488	1.140
APGAR index	0.515	0.350	0.088	1.471	0.143	−0.177	1.207

** p < 0.01, ^*^p < 0.05.

The level of PPD is positively and significantly correlated with the length of labor and the duration of the administration of epidural analgesia. On the contrary, the correlation between PPD and the child's APGAR index is not significant. Finally, the duration of labor is positively and significantly correlated with the duration of the administration of epidural analgesia.

Results of the linear regression showed that the model composed by the variables regarding labor, delivery and birth characteristics explained 44% of the variance (see Table 4). Specifically, cesarean delivery (dummy variable: 0 = cesarean delivery; 1 = vaginal delivery), and the duration of the administration of epidural analgesia seem to positively affect the severity of PPD. On the contrary, the results showed the no-significant influences of the modality of labor (dummy variable: 1 = induced labor; 0 = spontaneous labor) and the duration of labor, or the child's APGAR index, in affecting the severity of PPD.

### The Stronger Predictors of PPD Condition

Finally, a multiple regression was conducted to explore which of the significant risk factors found in the previously reported analyses make meaningful contributions to the overall prediction of the severity of PPD, which are: age, anxiety, depression, parental relationship, romantic relationship, prenatal attachment, typology of delivery, and epidural analgesia. All these predictors were entered at the first step, using a stepwise method.

Results showed that the model is composed of only four variables, which explain 61% of the PPD total score variance. Specifically, prenatal attachment to child entered into the equation in Step 1, which accounted for the greatest portion of the variance in PPD scores. The duration of the administration of epidural analgesia entered in Step 2, which contributed an additional 10% of variance. The anxiety score entered in Step 3, which contributed an additional 6%. Finally, the quality of romantic relationships entered in Step 4, which contributed an additional 3%. In Table 5, all statistical results are reported.

**Table 5:** Summary of the linear regression analysis using stepwise method with all risk significant factors as independent variables for PPD score.

**Predictors**	***B***	***SE***	***B St*.**	***t***	***p***	**Δ*R^**2**^***	***95% CI***	
**Step 1**								
Prenatal attachment	−0.326	0.030	−0.660	−11.063	0.000	0.435	−0.385	−0.268
**Step 2**								
Epidural analgesia	0.673	0.117	0.385	5.779	0.000	0.528	0.443	0.903
**Step 3**								
Anxiety	0.174	0.038	0.277	4.628	0.000	0.582	0.100	0.248
**Step 4**								
Partner relationship	−0.141	0.041	−0.177	−3.419	0.001	0.608	−0.223	−0.060

## Discussion

Pregnancy and the postpartum period is a delicate moment in a woman's life, characterized by biological, psychological and social change, during which women are at increased risk of emotional vulnerability and depressive symptomatology (1). This is especially true for nulliparous women, who, in addition to the normal psychic, psychological and relational changes typical of the pregnancy period, are dealing with more specific challenges, such as the transition to motherhood, or the reorganization of mental self-representation. At the first pregnancy, all these changes and reorganizations can represent challenges which are particularly relevant for women, and the presence of problems that prevent them from reaching a good transition to motherhood puts them at greater risk of developing a subsequent depressive symptomology. According to literature, primiparous mothers present an enhanced risk for depression in the postpartum period (17, 18) and more severe effects of depression in early interactions with their infants (22), due to their inexperience compared to multiparous mothers.

In literature, several factors have been found related to PPD, and we believe that the complex interplay of these can be the cause of greater vulnerability in nulliparous women (27–29). For this reason, the main purpose of this study was to explore the role that several sets of variables, such as socio-demographic, individual, relational, and related to delivery characteristics, separately considered, play as risk factors for the onset of postpartum depression in nulliparas. However, to date, no studies have jointly analyzed all these different risk factors. Consequently, the main and second purpose was to verify which have a more significant influence when they are considered together, in order to identify the more important risk factors.

Overall, our results show that, in reference to socio-demographic characteristics, only the age of women was a significant predictor of PPD scores. Older women seem have a higher probability of developing a depressive symptomatology 1 month after childbirth. On the contrary, in line with Roomruangwong's results (29), we found no significant influences of the level of education, employment status, the length of the romantic relationship, or planned pregnancy.

In reference to psychopathological characteristics, our results showed a strong association between prenatal psychopathology and the possibility to develop PPD. According to previous studies (28, 29, 53), nulliparous with a high level of anxiety and depression during pregnancy tend to develop PPD symptomatology more than women with a low level of these characteristics before delivery. Not surprisingly, due to the high association between antenatal and post-partum depression, the last edition of the DSM (9) proposed the term “peripartum onset” to indicate the depressive episode occurring during the pregnancy and the first 4 weeks after delivery.

Regarding the relational variables, our results indicated that only the prenatal attachment to child and the quality of romantic relationship affect the level of PPD (30, 32). In particular, prenatal attachment, defined as the emotional bond experienced by the parent toward the infant (54), seems to play a very important role in PPD, given that it involves the maternal disposition toward fetus and a protection attitude toward baby (55, 56). New research has suggested that this type of attachment is an indicator of the caregiving system (56, 57).

Interestingly, it is not the length of the relationship but its quality that better influences the security and well-being of nulliparous women. In has been widely recognized that the quality of the romantic relationship is a strong protective factor in the life-span (58, 59), and this could play an important role, especially in certain periods of life, such as the puerperium (60). During the transition to parenthood, the couple relationship may be negatively affected during pregnancy, given that birth of the first child leads to partnership re-organization of responsibilities and reciprocal routines, thus decreasing relationship quality (61). Therefore, a high quality of couple relationship constitutes a protective factor, and a low quality of romantic relationship constitutes a significant risk factor for the development of psychological diseases, such as a higher probability to develop depressive symptoms (62) and other forms of psychopathology (63).

The quality of relationship with parents also affects the level of PPD. In reference to a woman's relationship with her parents, this result is convergent with previous studies that have found that the bond quality to mother significantly influences a woman's well-being during pregnancy and after delivery, considerably reducing the risk to develop a PPD condition (64). Tani et al. (65, 66) have shown that the security of attachment to mother affects the quality of prenatal attachment and the consequent ability of the new mother to be sensitive to her newborn's needs, promoting spontaneous caregiving and attachment behavior. Maternal perceived support could also be considered a relevant protector factor: women who reported a high level of maternal social support showed a lower level of PPD symptomatology after labor (31). However, although in line with previous studies, this study expands on existing literature, showing the protective role of both maternal and paternal relationships on nulliparous women postnatal depression. It is reasonable to suppose that quality of relationship with parents is relevant during the first pregnancy, given that it can help women in the transition to motherhood and the assumption of the maternal role.

Finally, regarding labor factors, our data showed that a longer duration of the administration of epidural analgesia could be considered significant predictors of PPD in nulliparous. As suggested by previous studies, these factors can negatively influence the quality of the birth experience, fostering the possibility of developing a depressive mood, while an easier childbirth experience can act as a protective factor during the vulnerable period after delivery (31, 67–69). Regarding delivery factors, our findings suggest that emergency cesarean delivery affects post-partum depression in the nulliparous. This seems to confirm that women who express a strong desire to have a natural childbirth during pregnancy, but who must undergo cesarean section, are more prone to risk of postpartum depression (70).

Finally, our results highlighted no significant influences of the other variables considered, such as the length and the modality of labor and the APGAR index. In our sample, newborns showed good general condition. In fact, only three children (1.9%) obtained a score lower than 7, and no scores lower than six.

The second aim of this study was to explore which, among the above considered risk factors, were the stronger ones. Our analysis showed that, despite the marginal role of variables linked to clinical delivery difficulties, prenatal women's psychopathological characteristics and poor quality of couple relationship, prenatal attachment to child was the most important predictor of PPD, which, by itself, explains almost all the variance of the tested model.

Prenatal attachment expresses the first internalized representation that a pregnant woman develops of her unborn child and the emotional tie with him/her. Therefore, it is reasonable to hypothesize that a nulliparous with a good representation of her future child could experience her pregnancy with positive feelings, and that this, in turn, could improve the transition to her parenting role and well-being.

Extensive literature has shown that women who felt more affection toward their unborn children have more compliance with health practices during pregnancy (71, 72), present less clinical complications during delivery, and show less difficulty, more confidence and better adjustment in assuming the new parenting role (73). Moreover, it has been found that prenatal attachment to the fetus plays a positive and significant role in promoting more adequate mother-child interactions (61) and spontaneous caregiving and attachment behavior after childbirth (65). Therefore, it is not surprising that the mother's prenatal attachment to child could represent a key element to improving the health of the mother and child, the outcome being a significant protective factor to prevent PPD onset.

Despite the doubtless interest of these results, there are some limitations to this study. The first is the inclusion criteria. Only nulliparous women with no risk pregnancy, no twins, or previous miscarriage or abortion, were included in this study. Therefore, future research should extend study to samples of women who have twin pregnancies, or with high-risk pregnancies. A second limitation is that our study only investigated the role of relational variables connected to the women's close relationship network, not considering the role that relationships with gynecologists, midwives, and medical and nursing staff play in influencing the mothers' delivery experience during pregnancy and childbirth, and postpartum depression outcomes. An important direction for future research would be to extend this study to other aspects of the women's social network. Providing these additional data could allow us to develop specific and targeted preventive interventions for PPD, and a better understanding of which variables could be important in women with a more complicated history of pregnancy. A third limitation to this study is that PPD was assessed at 1 month post birth. Although it has been recognized that the vulnerability for depression continues for 6 months after delivery (14–16), according to DSM-5, depression with postpartum onset is an episode of major depression that occurs in the 4 weeks following delivery. In any case, further research should extend the follow-up to a longer period of time, for example, 4–6 months of the child's life. It is necessary to underline that the number of comparisons between the methods of birth is very different. In future research, it would be appropriate to have a more homogeneous sample, to compare PPD both after cesarean and after spontaneous or induced vaginal birth.

Finally, further studies with larger samples are desirable to replicate our findings.

Overall, the results of the present study have great relevance for clinical practice. Perinatal depression is an important public health issue with aftermath for mothers, children, and families. This is especially true for women during the first pregnancy, because of their greater emotional vulnerability. Timely screening and appropriate treatment are needed to prevent unnecessary suffering. Until now, screening and preventive health programs have given attention to medical and clinical risk factors. However, our results stress the importance of new focus on more comprehensive care and wellness, suggesting the promotion of mothers' and children's health in perinatal phases by expanding initiatives in clinical practice to additional behavioral and psychosocial screenings. Besides the importance of an early detection of depressive and anxious symptoms in nulliparous women, the present results highlight the relevant role played by relational factors, such as the quality of prenatal attachment to child, the relationship with partner, and the relationship with parents. All these relationships need to be taken into consideration to guarantee a more positive outcome toward the transition to motherhood. Moreover, it may be important to provide more information to women about the various possibilities of labor and delivery: what they are, what consequences they have, not only on the health of the mother and child, but also on the mother-child relationship, in order to allow women to have a more comprehensive understanding of these important aspects.

In conclusion, we believe that these results could have relevant clinical and social implications. Understanding the most important relevant risk factors for PPD is essential for identifying nulliparous pregnant women at risk and promptly intervening with specific preventive health care programs.

## Data Availability Statement

The dataset analyzed during the current study is available from the corresponding author on reasonable request.

## Ethics Statement

The study was conducted in accordance with the guidelines for the ethical treatment of human participants of the Italian Psychological Association. All subjects gave written informed consent in accordance with the Declaration of Helsinki. The protocol was approved by the Ethical Committee of Azienda USL 4 Prato, Italy (no 780/2013).

## Author Contributions

MS participated in the development of protocol and analytical framework for the study. Moreover, she contributed to the drafting of the introduction and discussion sections of the manuscript. LP had primary responsibility for protocol development, patient screening, and participant's enrolment. Moreover, she performed the data analysis and contributed to draft the Methods and Materials section. FP revised the manuscript critically for important intellectual content.

### Conflict of Interest

The authors declare that the research was conducted in the absence of any commercial or financial relationships that could be construed as a potential conflict of interest.
